# Dopaminergic medication impairs feedback-based stimulus-response learning but not response selection in Parkinson's disease

**DOI:** 10.3389/fnhum.2014.00784

**Published:** 2014-10-02

**Authors:** Andrew Vo, Nole M. Hiebert, Ken N. Seergobin, Stephanie Solcz, Allison Partridge, Penny A. MacDonald

**Affiliations:** ^1^The Brain and Mind Institute, University of Western OntarioLondon, ON, Canada; ^2^Department of Psychology, University of Western OntarioLondon, ON, Canada; ^3^Department of Physiology and Pharmacology, University of Western OntarioLondon, ON, Canada; ^4^Schulich School of Medicine and Dentistry, University of Western OntarioLondon, ON, Canada; ^5^Department of Clinical Neurological Sciences, University of Western OntarioLondon, ON, Canada

**Keywords:** Parkinson's disease, dopamine, cognitive impairment, learning, stimulus-response

## Abstract

Cognitive dysfunction is a feature of Parkinson's Disease (PD). Some cognitive functions are impaired by dopaminergic medications prescribed to address the movement symptoms that typify PD. Learning appears to be the cognitive function most frequently worsened by dopaminergic therapy. However, this result could reflect either impairments in learning (i.e., acquisition of associations among stimuli, responses, and outcomes) or deficits in performance based on learning (e.g., selecting responses). We sought to clarify the specific effects of dopaminergic medication on (a) stimulus-response association learning from outcome feedback and (b) response selection based on learning, in PD. We tested 28 PD patients on and/or off dopaminergic medication along with 32 healthy, age- and education-matched controls. In Session 1, participants learned to associate abstract images with specific key-press responses through trial and error via outcome feedback. In Session 2, participants provided specific responses to abstract images learned in Session 1, without feedback, precluding new feedback-based learning. By separating Sessions 1 and 2 by 24 h, we could distinguish the effect of dopaminergic medication on (a) feedback-based learning and response selection processes in Session 1 as well as on (b) response selection processes when feedback-based learning could not occur in Session 2. Accuracy achieved at the end of Session 1 were comparable across groups. PD patients on medication learned stimulus-response associations more poorly than PD patients off medication and controls. Medication did not influence decision performance in Session 2. We confirm that dopaminergic therapy impairs feedback-based learning in PD, discounting an alternative explanation that warranted consideration.

## Introduction

Parkinson's disease (PD) is a common movement disorder, though cognitive abnormalities are now recognized. These non-motor, cognitive symptoms are a leading cause of poor quality of life in PD (Schrag et al., 2000; Barone et al., 2009). Dopaminergic medications, such as L-3,4-dihydroxyphenylalanine (L-dopa) or dopamine receptor agonists, prescribed to address motor symptoms of tremor, bradykinesia, and rigidity, seem to improve some cognitive functions and to worsen others (Cools, 2006; MacDonald and Monchi, 2011). The paradoxical effects of medication on different aspects of cognition have been explained by differences in endogenous dopamine concentrations in the brain regions that underlie them.

In PD, movement abnormalities appear, and a diagnosis is confirmed, when degeneration of dopamine-producing cells of the substantia nigra (SN) is sufficient to seriously restrict dopamine supply to its efferent, the *dorsal* striatum (DS) (Kish et al., 1988). In contrast, dopamine-producing cells in the ventral tegmental area (VTA) are relatively spared and dopamine supply to the *ventral* striatum (VS), along with limbic and frontal cortices, is better preserved (Haber and Fudge, 1997). The striatum is the input structure for a collection of subcortical nuclei that are broadly implicated in movement regulation and increasingly in cognitive functions. The DS includes the bulk of the caudate nucleus and the putamen. The *VS*, comprising the nucleus accumbens and the most ventral portions of caudate and putamen, is considered separately from the DS because these regions have distinct dopaminergic afferents (Voorn et al., 2004; Wickens et al., 2007), vascular supplies (Feekes and Cassell, 2006), and functions (Cools, 2006; MacDonald and Monchi, 2011). As the pathophysiology would predict, dopaminergic medications substantially improve DS-mediated motor and cognitive symptoms (Cools, 2006; MacDonald and Monchi, 2011; Colzato et al., 2012). However, in PD, these medications appear to worsen cognitive operations performed by VTA-innervated brain regions presumably due to dopamine overdose of these dopamine-replete brain regions (Gotham et al., 1988; Cools et al., 2001; Cools, 2006; MacDonald and Monchi, 2011).

A survey of the literature suggests that learning, in various forms, is the cognitive function most commonly worsened by dopaminergic medication. Studies that have tested PD patients on relative to off medication have reported impairments in probabilistic associative (Torta et al., 2009; Jahanshahi et al., 2010), sequence (Feigin et al., 2003; Ghilardi et al., 2007; Seo et al., 2010; Tremblay et al., 2010), and stimulus-reward reversal learning (Swainson et al., 2000; Cools et al., 2002; Tomer et al., 2007; Graef et al., 2010; MacDonald et al., 2013a), as well as explicit abstract figure and list learning (MacDonald et al., 2013b), stimulus-stimulus facilitation (MacDonald et al., 2011), and learning from negative feedback (Frank et al., 2004). However, investigations often fail to separately assess the acquisition of associations among stimuli, responses, and outcomes, from processes of response selection that rely on these learned associations and that are used to measure new learning (McDonald and White, 1993; Jessup and O'doherty, 2011). For example, typical stimulus-response learning paradigms proceed as follows: (a) a stimulus is presented and participants decide among a set of responses, (b) feedback about the accuracy of the response is provided, through which stimulus-response associations are learned. Stimulus-response association learning is estimated by measuring the accuracy of stimulus-specific response selection and enactment. Impairment in either learning or in using learned associations to decide among responses could yield poor performance in these standard learning paradigms.

Atallah et al. (2007) demonstrate this point quite elegantly. An extensive literature existed linking DS to learning associations among stimuli, responses, and rewards (Yin and Knowlton, 2006; Ashby et al., 2007). However, noting the confound outlined above, Atallah and colleagues sought to separate the acquisition of associations from performance based on this learning. In a Y-maze task using odor cues, they observed impairment in rats' abilities to consistently select a rewarded vs. unrewarded arm in animals receiving infusions of inhibitory gamma-amino butyric acid (GABA) agonist to DS compared to a saline solution during the learning phase of the experiment. At first blush, this seemed to suggest that animals receiving inhibitory infusions to DS were learning associations between odor cues and rewards more poorly. When both groups were later tested once the infusions were stopped, however, both experimental and control groups performed the selection task similarly. This demonstrated that associations were learned equally well for both experimental and control (i.e., saline-infused) groups during Session 1 and suggested that inhibition of DS impaired the animal's ability to use learned associations to perform selections reliably. To complement this interesting finding, in another experiment, they found that GABA infusions to DS at test phase resulted in impaired selection performance compared to saline infusions to DS, although both groups had previously shown identical learning of these odor-reward associations during the training phase. Taken together, these results challenged the widely held notion that DS mediates learning and instead suggested a more specific role for DS in performance based on learning.

**Figure 1:**
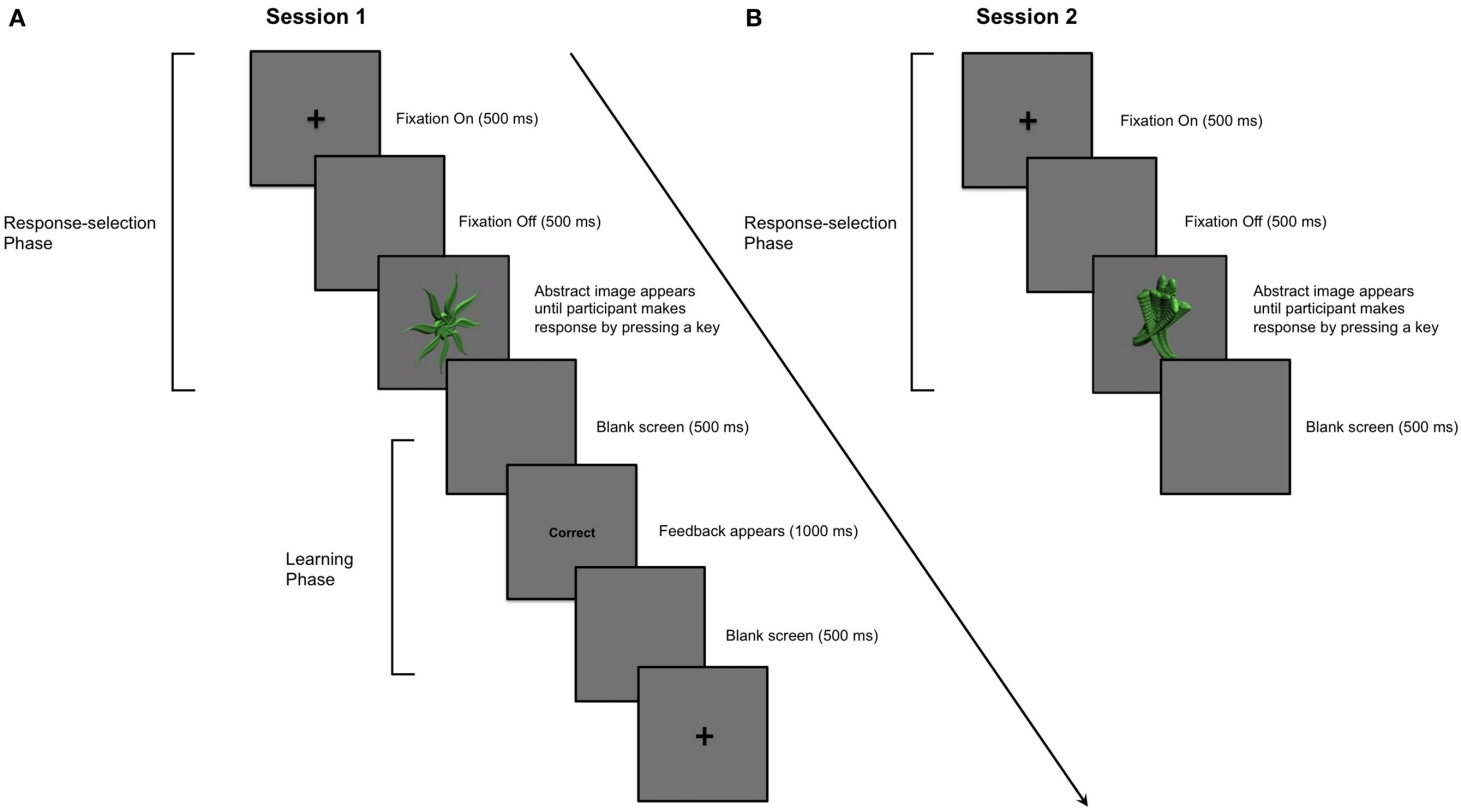
**Example of a single trial in Sessions 1 and 2. (A)** Session 1: PD patients and aged-matched controls learned to associate 9 abstract images with a key-press response. A trial proceeded as follows: (i) a cross appeared in the center of a computer screen for 500 ms; (ii) a blank screen was presented for 500 ms; (iii) an abstract image was presented in the center of the computer screen until the participant entered his or her key-press response using the “1,” “2,” or “3” numeric keys; (iv) the image disappeared before feedback, either the word “Correct” or “Incorrect,” was presented for 1000 ms in the center of the screen; (v) a blank screen was presented for 500 ms before the next trial began. An abstract image was presented and participants provided his or her key-press response before feedback was presented. **(B)** Session 2: Stimulus-specific key-press responses for stimuli learned in Session 1 were performed in the absence of feedback a day later. The parameters for each trial in Session 2 were otherwise identical to those in Session 1.

We note that the literature implicating dopaminergic medication in learning impairment in PD similarly warrants reconsideration. The specific aim of the present study was therefore to investigate the effect of dopaminergic medication in PD on stimulus-response learning versus response performance processes. In Session 1, PD patients and healthy age- and education-matched controls first learned to associate abstract images and specific key-press responses through outcome feedback. Session 1 constituted a typical stimulus-response learning study in which processes of stimulus-response association learning and response selection performance were confounded. In Session 2, participants were asked to make the specific key-press responses to abstract images that they had learned in Session 1. However, no feedback regarding the accuracy of responses was provided in Session 2, precluding new feedback-based learning. Figures 1A,B illustrate how trial structures in Sessions 1 and 2 differ from one another in only one regard—the provision of outcome feedback.

**Figure 2:**
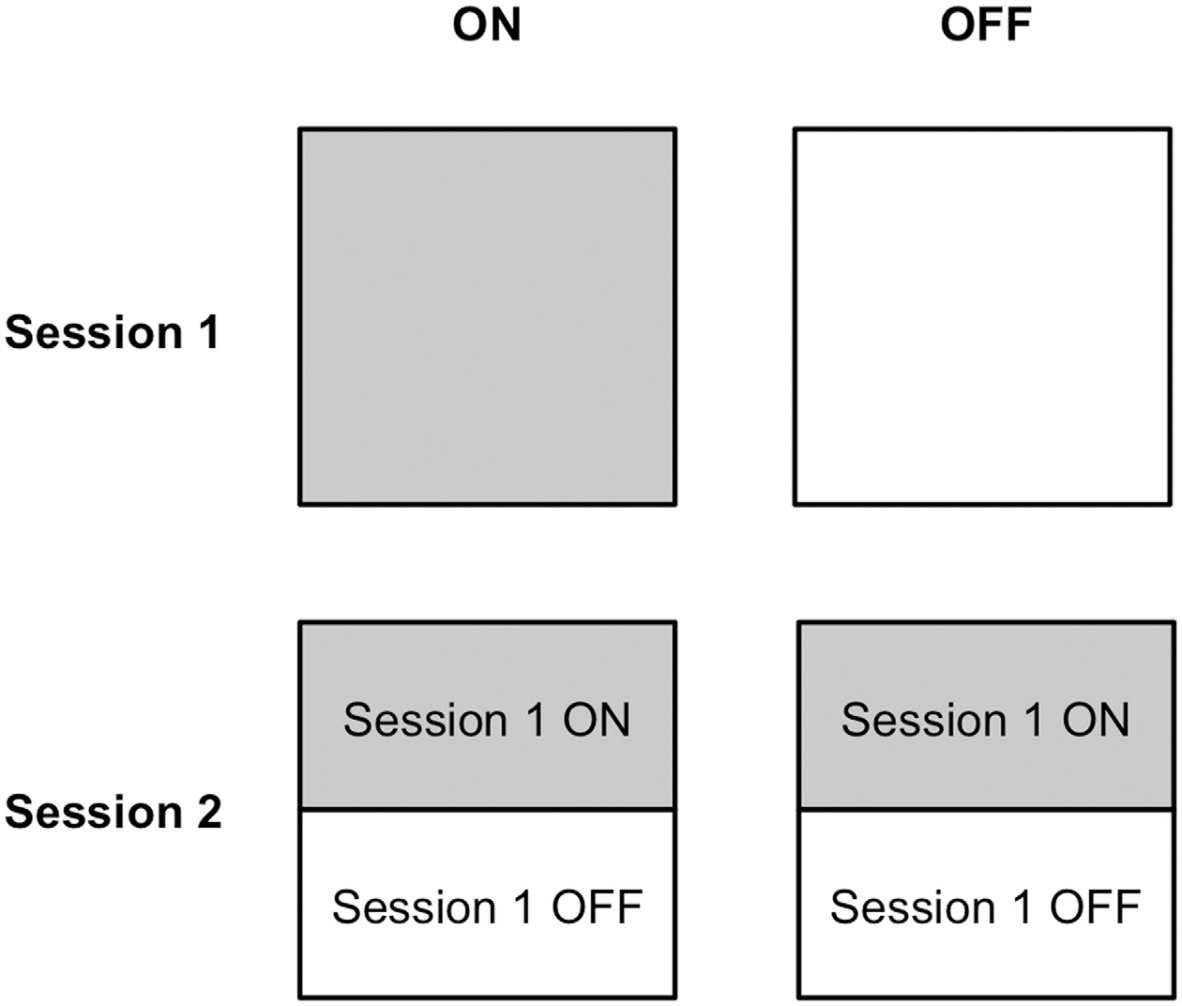
**Medication status assignment of participants in Sessions 1 and 2**. Half of participants completed stimulus-response learning on medication and the other half performed stimulus-response learning off medication in Session 1. Half of the participants in each of the ON and OFF medication groups in Session 2 had learned stimulus-response associations on medication in Session 1 and the other half had learned stimulus-response relations off medication.

Half of the PD patients completed Session 1 on medication, whereas the other half learned stimulus-response associations via feedback off medication. Similarly, half of the PD patients performed Session 2 on and the other half off dopaminergic medication. Because performance in Session 2 depended upon learning in Session 1, and we expected that medication status could influence learning in Session 1, we made two design choices to mitigate carry-over effects. First, we implemented a learning criterion in Session 1 to ensure that all participants achieved a similar level of stimulus-response association strength, without establishing overlearned relations. Second, we ensured that each on and off group in Session 2 was composed of an equal number of participants who had learned stimulus-response associations in Session 1 on compared to off medication. Please see Figure 2 to understand the design of this experiment.

## Materials and methods

### Participants

Thirty-three PD patients and 36 age- and education-matched healthy controls participated in the experiment. All PD patients were previously diagnosed by a licensed neurologist, had no coexisting diagnosis of dementia or another neurological or psychiatric disease, and met core assessment criteria for surgical interventional therapy and the UK Brain Bank for the diagnosis of idiopathic PD (Hughes et al., 1992). No PD patients were treated with deep brain stimulation or other neurosurgeries. Control participants were free of neurological and psychiatric illnesses. All PD patients and no controls were treated with dopaminergic therapy. This study was approved by the Health Sciences Research Ethics Board of the University of Western Ontario and the Ethics Review Board of the Sudbury Regional Hospital. All participants provided informed written consent to the approved protocol before beginning the experiment, according to the Declaration of Helsinki (World Medical Association, 2013).

Participants abusing alcohol, prescription, or street drugs, or taking cognitive-enhancing medications including Donepezil, Galantamine, Rivastigmine, Memantine, or Methylphenidate were excluded from participating. Five PD patients and four control participants did not reach a pre-set learning criterion (see Section Design and Procedure) in Session 1. They were not invited to participate in Session 2 and therefore their data were not included in our analyses. Consequently, we included 28 PD patients (21 males) and 32 control participants (11 males) in our analyses.

The motor sub-scale of the Unified Parkinson's Disease Rating Scale (UPDRS) was scored by a neurologist with sub-specialty training in movement disorders (Penny A. MacDonald) to assess the presence and severity of disease for all PD patients both on and off dopaminergic medication. Control participants were also screened to rule out undiagnosed neurological illness, PD in particular. In addition, all participants completed a battery of standardized cognitive and affective tests to rule out significant cognitive impairments, depression, or anxiety.

Table 1 presents mean group demographics, as well as affective and cognitive screening scores for all patients along with these measures for their matched controls. UPDRS motor subscale scores on and off usual dopaminergic medication, daily doses of dopaminergic therapy in terms of L-dopa equivalents, and mean duration of PD are also presented in Table 1. Calculation of daily L-dopa equivalent dose for each patient was based on the theoretical equivalence to L-dopa (Evans et al., 2004) as follows: L-dopa dose + L-dopa × 1/3 if on entacapone + bromocriptine (mg) × 67 + ropinerole (mg) × 20 + pergolide (mg) × 100 + apomorphine (mg) × 8. There were no significant demographic differences between PD patients and controls (Table 1). Screening cognitive measures confirmed that no participants suffered significant cognitive impairment (Table 1).

**Table 1:** **Demographic, clinical information, and screening cognitive and affective measures for Parkinson's disease patients and controls**.

**Group**	***N***	**Age**	**Edu**	**Duration**	**L-dopa**	**DA**	**UPDRS ON**	**UPDRS OFF**	**ANART**	**BDI-II**	**BAI**	**Apathy**	**MOCA**
**SESSION 1**													
PD	28	65.11 (1.30)	15.32 (0.53)	5.93 (0.76)	549.32 (64.62)	13	–	–	126.00 (0.48)	9.14 (0.90)	9.50 (1.30)	11.21 (0.92)	26.00 (0.48)
ON	15	66.07 (2.09)	15.27 (0.74)	5.33 (1.18)	532.60 (93.39)	5	13.00 (1.23)	–	123.27 (1.58)	9.80 (1.37)	11.27 (2.06)	11.00 (1.04)	25.53 (0.78)
OFF	13	64.00 (1.44)	15.38 (0.77)	6.62 (0.93)	568.62 (91.85)	8	–	13.81 (1.28)	127.08 (1.40)	8.38 (1.13)	7.46 (1.34)	11.46 (1.63)	26.54 (0.51)
Control	32	63.13 (1.44)	14.41 (0.53)	–	–	–	–	–	123.78 (1.21)	4.94 (0.74)	4.59 (1.11)	10.09 (0.89)	27.31 (0.38)
ON	16	63.50 (2.49)	14.63 (0.75)	–	–	–	–	–	122.81 (1.90)	4.56 (1.05)	5.69 (1.85)	10.38 (1.36)	27.13 (0.46)
OFF	16	62.75 (1.53)	14.19 (0.77)	–	–	–	–	–	124.75 (1.52)	5.31 (1.06)	3.50 (1.22)	9.81 (1.18)	27.50 (0.61)
**SESSION 2**													
PD	28	65.11 (1.30)	15.32 (0.53)	5.93 (0.76)	549.32 (64.62)	13	–	–	125.04 (1.11)	9.14 (0.90)	9.50 (1.30)	11.21 (0.92)	26.00 (0.48)
ON	15	64.20 (1.94)	15.40 (0.70)	5.47 (0.98)	480.67 (66.12)	8	12.50 (1.18)	–	124.40 (1.32)	9.33 (1.40)	7.73 (1.83)	11.13 (1.36)	25.53 (0.75)
OFF	13	66.15 (1.70)	15.23 (0.82)	6.46 (1.22)	628.54 (115.65)	5	–	16.15 (1.78)	125.77 (1.89)	7.77 (1.24)	6.62 (1.34)	12.00 (1.10)	26.54 (0.57)
Control	32	63.13 (1.44)	14.41 (0.53)	–	–	–	–	–	123.78 (1.21)	3.94 (0.69)	2.59 (0.67)	10.13 (0.87)	27.31 (0.38)
ON	17	61.76 (2.49)	13.88 (0.88)	–	–	–	–	–	122.24 (1.68)	4.59 (1.07)	3.35 (1.14)	11.59 (1.21)	27.18 (0.57)
OFF	15	64.67 (1.20)	15.00 (0.52)	–	–	–	–	–	125.53 (1.69)	3.20 (0.83)	1.73 (0.57)	8.47 (1.14)	27.47 (0.51)

Values are presented as group means (SEM). Screening cognitive and affective measures were completed by PD patients on medication unless indicated otherwise. Control participants did not receive dopaminergic medication during any session of the experiment. Their data are presented to correspond to the ON–OFF order of the patient with Parkinson's disease to whom they were matched. Edu, years of education; Duration, years since diagnosis of PD; L-dopa, daily L-dopa equivalent dose in mg; DA, number of PD patients taking DA agonists; UPDRS ON, Unified Parkinson's Disease Rating Scale motor score on medication; UPDRS OFF, Unified Parkinson's Disease Rating Scale motor score off medication; ANART IQ, National Adult Reading Test (Nelson and Willison, 1991) IQ Estimation; BDI-II, Beck Depression Inventory II score measured for PD patients and for matched control participants; BAI, Beck Anxiety Inventory I score measured for PD patients and for matched control participants; Apathy, Apathy Evaluation Scale score measured for PD patients and for matched control participants; MOCA, Montreal Cognitive Assessment measured for PD patients and for matched control participants.

### Apparatus

The experiment was conducted on a 14.0″ widescreen laptop (Lenovo T420) running at a resolution of 1600 × 900 on the Windows 7 operating system. The screen was placed at a distance of approximately 50 cm in front of participants and angled for optimal viewing.

### Stimuli

**Figure 3:**
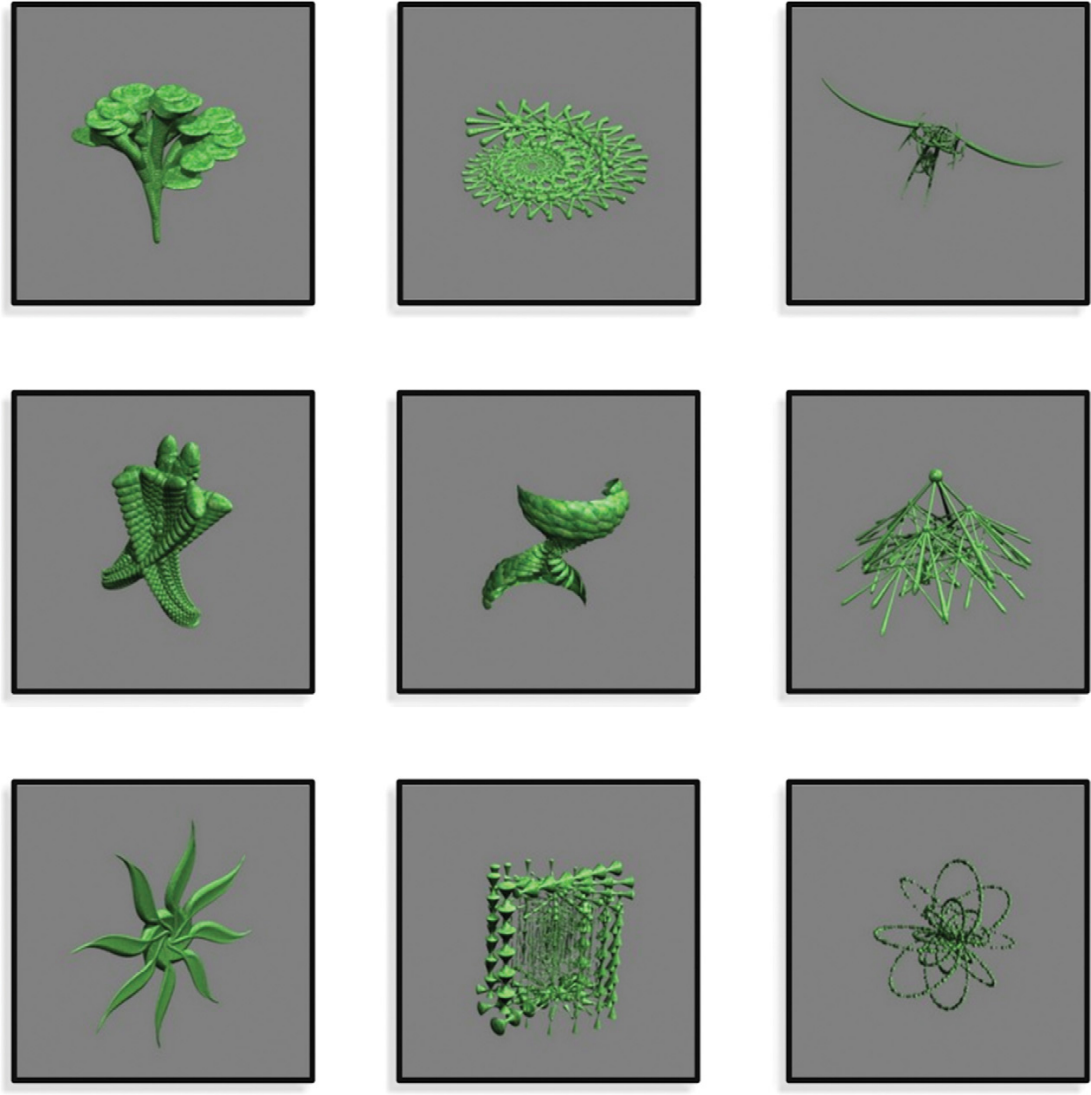
**Abstract images presented during Sessions 1 and 2**. Images were computer-generated with *GroBoto* (Braid Art Labs, Colorado Springs, USA).

The stimuli used during the experiment consisted of abstract images that were computer-generated with *GroBoto* (Braid Art Labs, Colorado Springs, USA). Nine abstract images were used in the experiment (Figure 3).

### Design and procedure

All patients with PD participated in two experimental sessions conducted over consecutive days, as did their age- and education-matched healthy controls. Half of the PD patients performed Session 1 on and the other half off dopaminergic medication. This assignment was random. Similarly, half of the PD patients performed Session 2 on and the other half off dopaminergic medication. For Session 2, half of the PD patients from the Session 1 ON condition were randomly assigned to the Session 2 ON condition whereas the other half were randomly assigned to the Session 2 OFF condition. Analogously, half of the PD patients from the Session 1 OFF condition were randomly assigned to the Session 2 ON condition whereas the other half were randomly assigned to Session 2 OFF condition. This is illustrated in Figure 2.

PD patients took their dopaminergic medication as prescribed by their treating neurologist during ON testing sessions, but abstained from taking L-dopa/carbidopa and entacapone for 12–18 h, and dopamine agonists, such as pramipexole, ropinirole, or pergolide, as well as amantadine, rasagiline, and selegiline for 16–24 h before beginning OFF testing sessions. Although control participants did not take dopaminergic medication during any session, their data were analyzed to correspond to the medication order of the PD patient to whom they were matched. Matching was performed at time of testing, prior to data analysis. This controlled for possible order, fatigue, and practice effects.

Before beginning Session 1, participants received 20 practice trials with different images from those employed during the main experimental sessions. In Session 1, participants performed a task during which they learned to associate abstract images with one of three key-press responses. On each trial, an abstract image appeared and remained in the center of the computer screen until the participant responded with a key press. Outcome feedback (i.e., “Correct” or “Incorrect”) was provided after every response and in this way, participants learned to associate each of the abstract images with the appropriate key-press response through trial and error.

All trials proceeded as follows: (i) a cross appeared in the center of a computer screen for 500 ms; (ii) a blank screen occurred for 500 ms; (iii) an abstract image was presented until the participant entered a key-press response, either “1,” “2,” or “3” keys; (iv) feedback, either “Correct” or “Incorrect,” was presented for 1000 ms; (v) a blank screen for 500 ms separated trials (Figure 1A).

Trials were organized into blocks. Each block consisted of 18 trials, with each of the nine abstract images occurring twice in random order. Three images were assigned to each of the “1,” “2,” and “3” numeric keys at the top of the keyboard and participants pressed these keys with their index, middle, and ring fingers, respectively. After each block, participants were provided with a percentage score, summarizing their learning performance. A minimum learning criterion of 74% on two successive blocks was required to complete Session 1. This ensured that similar learning was achieved by all participants and ensured that over-learning of associations did not occur.

Session 2 occurred the day after Session 1, approximately 24 h later for each participant. In Session 2, participants performed two blocks of 18 trials, in which the same 9 images studied during Session 1 were presented in random order, twice per block. Participants decided among and selected the key-press response that they had learned for each image in Session 1. No outcome feedback was provided to preclude new feedback-based learning. The parameters for each trial in Session 2 were otherwise identical to those in Session 1 (Figure 1B).

We expected that dopaminergic medication might have an effect on learning in Session 1. Performance in Session 2 depended on how well stimulus-response associations were learned in Session 1. To diminish any carry-over effects from Session 1, we (i) imposed a pre-set learning criterion of 74% in Session 1 and (ii) included an equal number of participants who learned on and an equal number of participants who learned off medication in Session 1, in each of the ON and OFF conditions in Session 2 (Figure 2).

### Behavioral measures

Efficiency of encoding stimulus-response associations across Session 1 was estimated by a mean improvement score that describes the change in percent correct performance per block across Session 1. This score was calculated as follows: [Block_1_ + (Block_2_ − Block_1_) + (Block_3_ − Block_2_) + (Block_4_ − Block_3_) +… (Block_N_ − Block_N_ − 1)] ÷ Number of Blocks (N). In Session 2, decision making based on previously-learned associations was measured with an adjusted-savings score, calculated as follows: average accuracy in Session 2 ÷ accuracy in the last block of Session 1. Anticipating that Group (PD patients vs. Controls) and Medication status (ON vs. OFF) might affect learning, we implemented several measures to reduce the variability in the degree to which stimulus-response associations were learned by all (i.e., compelling all participants to reach a criterion of 74% and ensuring that the groups in Session 2 were entirely balanced with respect to learning conditions in Session 1). In the event that comparable stimulus-response learning was not achieved by all groups, however, the use of an adjusted-savings score was intended to correct for subtle variability among participants in stimulus-response association learning. This allowed us to examine how PD and dopaminergic medication affected recall and enactment of previously-learned stimulus-specific responses independently of how these factors affected stimulus-response association learning *per se*. Higher improvement scores indicated more efficient learning, and higher adjusted-savings scores indicated superior retention of learned associations and decision-making performance based on prior learning. Separate One-Way analyses of variance (ANOVAs) were conducted on stimulus-response association learning estimates and measures of response selection performance between Groups (PD patients vs. Controls) and across Medication status (ON vs. OFF).

## Results

**Figure 4:**
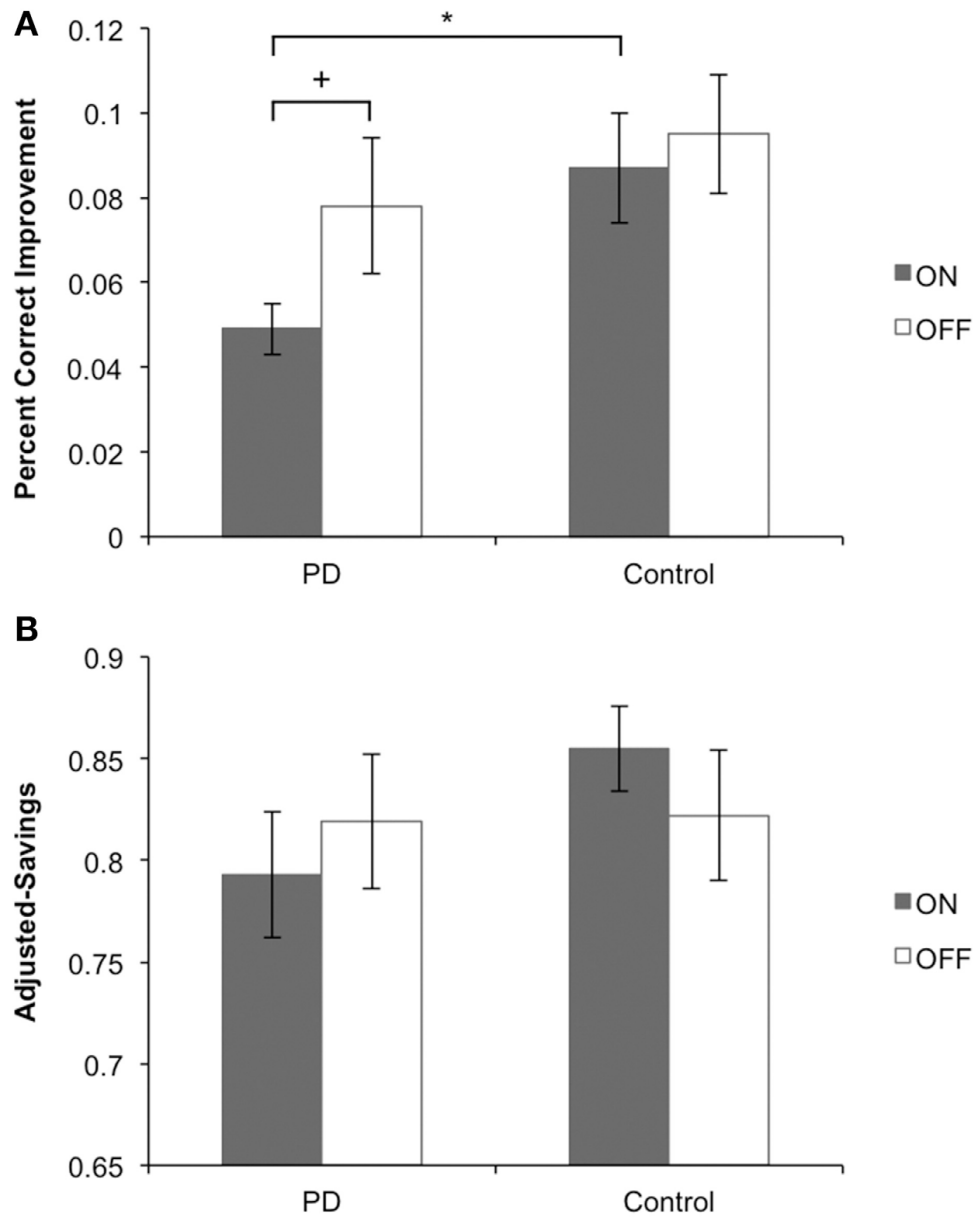
**Main behavioral findings for Sessions 1 and 2**. **(A)** Session 1: PD patients on medication learned stimulus-response associations more poorly than their matched controls and more poorly than PD patients off medication. PD patients off medication learned equally well as their matched controls. **(B)** Session 2: PD patients on medication performed stimulus-response associations equally well to PD patients off medication. In each medication condition, PD patients performed equally well as their matched controls.

Main findings for Sessions 1 and 2 are presented in Figures 4A,B, respectively. Other behavioral data for Sessions 1 and 2 are presented in Table 2.

**Table 2:** **Behavioral results for Parkinson's disease patients and controls**.

	**Session 1**			**Session 2**
	**Initial score**	**Final score**	**Blocks to criterion**	**Absolute savings**
**PD**				
ON	0.352 (0.021)	0.822 (0.015)	20.1 (2.34)	0.676 (0.030)
OFF	0.372 (0.031)	0.842 (0.020)	18.3 (3.730)	0.662 (0.030)
**CONTROL**				
ON	0.351 (0.030)	0.826 (0.014)	14.8 (3.173)	0.722 (0.018)
OFF	0.385 (0.094)	0.858 (0.012)	13.4 (2.137)	0.689 (0.030)

All values reported are means (SEM). Initial Score is the score achieved on the first block of Session 1. Final Score is the score achieved in the final block of Session 1. Blocks to Criterion is the total number of blocks needed to reach the learning criterion in Session 1. Absolute Savings in Session 2 was calculated as the average score in Session 2.

### Session 1: learning stimulus-response associations via outcome feedback

We performed a One-Way ANOVA on improvement scores, with Group (PD patients vs. Control) as the between-subject factor, for ON and OFF medication conditions separately. PD patients learned significantly more poorly than matched control participants in the ON session [*F*_(1, 29)_ = 6.587, MSE = 0.011, *p* = 0.016], whereas PD patients and matched controls learned equally well off medication [*F*_(1, 27)_ = 0.659, MSE = 0.002, *p* = 0.424]. We performed another One-Way ANOVA on improvement scores with Medication status (ON vs. OFF) as the between-subject factor in PD patients and controls separately. Consistent with findings from our first ANOVA, PD patients showed a statistical trend toward poorer learning of stimulus-response associations ON compared to OFF medication [*F*_(1, 26)_ = 3.081, MSE = 0.006, *p* = 0.077]. No differences were found for controls comparing pseudo-ON and pseudo-OFF medication sessions [*F*_(1, 30)_ = 0.207, MSE = 0.001, *p* = 0.652].

To rule out the possibility that PD patients and controls simply displayed differences in performance on the initial block with similar learning efficiency, we performed One-Way ANOVAs on the first block scores. PD patients did not differ from their matched-control participants for either ON [*F*_(1, 29)_ = 0.001, MSE = 0.000, *p* = 0.978] or OFF [*F*_(1, 27)_ = 0.101, MSE = 0.001, *p* = 0.753] medication conditions. Further, no differences were found for PD patients comparing ON and OFF medication sessions [*F*_(1, 26)_ = 0.292, MSE = 0.003, *p* = 0.594] and controls comparing pseudo-ON and –OFF medication sessions [*F*_(1, 30)_ = 0.681, MSE = 0.010, *p* = 0.416].

Analogously, we performed a One-Way ANOVA on final block scores to ensure that PD patients and controls reached a comparable level of learning performance in Session 1. PD patients did not differ from their matched-control participants for either ON [*F*_(1, 29)_ = 0.042, MSE = 0.000, *p* = 0.839] or OFF [*F*_(1, 27)_ = 0.487, MSE = 0.002, *p* = 0.491] medication conditions. Further, no differences were found for PD patients comparing ON and OFF medication sessions [*F*_(1, 26)_ = 0.662, MSE = 0.003, *p* = 0.423] and controls comparing pseudo-ON and -OFF medication sessions [*F*_(1, 30)_ = 2.720, MSE = 0.008, *p* = 0.110].

### Session 2: performing stimulus-specific responses

For performance in Session 2, we conducted a One-Way ANOVA on adjusted-savings scores with Group (PD patients vs. Controls) as the between-subject factor, for ON and OFF medication conditions separately. PD patients OFF [*F*_(1, 30)_ = 2.58, MSE = 0.011, *p* = 0.120] and ON (*F* < 1) medication performed comparably to controls. Similarly, no ON–OFF differences were found for PD patients or controls (both *F* < 1).

Despite measures taken to mitigate the influence of learning in Session 1 on performance in Session 2, there remained the possibility for carry-over effects between Sessions. We therefore performed a 2 × 2 ANOVA on adjusted-savings scores with Group (PD patients vs. Control) and Medication (ON vs. OFF) as the between-subject factors, covaried with Medication during Session 1 (ON vs. OFF). There were no significant main effects of either Group [*F*_(1, 56)_ = 1.191, MSE = 0.015, *p* = 0.280] or Medication [*F*_(1, 56)_ = 0.015, MSE = 0.000, *p* = 0.904] conditions. Further, there was no significant interaction effect [*F*_(1, 56)_ = 1.002, MSE = 0.013, *p* = 0.321]. These results suggest that the measures taken to reduce the influence of Session 1 learning on Session 2 performance were successful in ensuring that despite any effect of group or medication in Session 1, all participants achieved a similar, pre-asymptotic level of stimulus-response association learning.

## Discussion

In Session 1, participants learned to associate stimuli and responses through outcome feedback. Learning was evidenced when participants correctly recalled the associations, selected, and enacted stimulus-specific responses. In Session 2, feedback was omitted, eliminating the possibility of further feedback-based learning Participants expressed what they had previously learned by enacting the stimulus-specific responses from Session 1 (see Figures 1A,B). Off dopaminergic medication, PD patients performed Sessions 1 and 2 normally compared to age-matched controls. In contrast, PD patients on dopaminergic medication learned stimulus-response relations in Session 1 more poorly than controls. Bolstering this finding further, PD patients on dopaminergic medication achieved the learning criterion in Session 1 less efficiently than PD patients off medication. In Session 2, PD patients on and off dopaminergic therapy performed equivalently. We interpret these results as evidence that PD patients learn stimulus-response associations normally at baseline and that dopaminergic medication impairs feedback-based learning but not recall of these associations, or response selection and enactment.

Stimulus-response learning paradigms often proceed in a single session as follows: (a) a stimulus is presented and participants decide among a set of responses, (b) feedback about the accuracy of the response is provided, through which stimulus-response associations are learned. Stimulus-response association learning is indexed by the accuracy of recalling, selecting, and enacting stimulus-specific responses. Impairment in either learning *per se* or response selection/enactment based on what has been learned could yield poor performance in this paradigm (Atallah et al., 2007). These data do not suggest that dopaminergic therapy actually impairs performance of stimulus-specific response selection processes, giving the appearance of deficient stimulus-response association learning (Atallah et al., 2007). This confound was addressed by employing a rigorous methodology in which we: (1) tested performance in a session where feedback-based learning was possible relative to one in which it was not, (2) equated trial structures in Sessions 1 and 2 so that they differed only in terms of the provision of outcome feedback, (3) prevented over-learning/ceiling performance by using a 74% accuracy criterion in Session 1 and introduced a 24 h delay between Sessions 1 and 2 to produce comparable average performance in each session in terms of accuracy (see Table 2), (4) mitigated carry-over effects from Session 1 to Session 2 by equating the learning achieved by all participants in Session 1 and including an equal number of PD patients in the ON and OFF conditions in Session 2 from ON and OFF conditions in Session 1 (see Figure 2), and (5) matched PD patients to healthy age-matched controls, analyzing control data to correspond to the ON–OFF order of their PD patient, eliminating the possibility that our findings owed simply to complex order effects.

These results support findings from previous studies in PD that report a medication-associated impairment in various forms of learning (Swainson et al., 2000; Cools et al., 2002; Feigin et al., 2003; Frank et al., 2004; Ghilardi et al., 2007; Tomer et al., 2007; Torta et al., 2009; Graef et al., 2010; Jahanshahi et al., 2010; Seo et al., 2010; Tremblay et al., 2010; MacDonald et al., 2011 but see Shiner et al., 2012; Smittenaar et al., 2012). Whereas motor symptoms and some cognitive functions are improved by dopaminergic medication, other cognitive processes are actually worsened (Cools, 2006; MacDonald and Monchi, 2011). These differential effects of dopaminergic therapy on individual cognitive functions likely owe to differences in endogenous dopamine in the brain regions that mediate them (Gotham et al., 1988; Cools et al., 2001; Cools, 2006; MacDonald and Monchi, 2011). At baseline, DS appears to be severely dopamine-depleted whereas VTA-innervated brain regions, including VS, limbic, and prefrontal cortices are relatively dopamine replete. Dopaminergic medication rectifies this dopamine deficit and ameliorates DS-mediated motor and cognitive functions (Feigin et al., 2001; Cools et al., 2003; Asanuma et al., 2006; Wu et al., 2009; MacDonald et al., 2011; Colzato et al., 2012). This appears to be at the expense of functions performed by VTA-innervated brain regions, which are worsened by medication, especially at earlier stages of PD (Cools, 2006; MacDonald and Monchi, 2011). Consistent with the dopamine overdose hypothesis and the findings presented here, learning has been ascribed to both VS (O'doherty, 2004; Shohamy et al., 2004, 2006; Reiss et al., 2005; Cools et al., 2007; Ghilardi et al., 2007; Seo et al., 2010; Tremblay et al., 2010; MacDonald et al., 2011) and limbic cortical regions (McDonald and White, 1993; Rodriguez, 2009). Indeed, in a recent study, we found that VS activity correlated with feedback-based stimulus-response learning in a procedure that was virtually identical to the one presented here (Hiebert et al., 2014). Finally, and further supporting our interpretation of our findings, even in healthy volunteers, administration of dopaminergic therapy has been shown to worsen learning (Mehta et al., 2001; Breitenstein et al., 2006).

Cognitive dysfunction is now an undisputed non-motor symptom of PD that leads to significant impairment in quality of life (Schrag et al., 2000; Barone et al., 2009). Whereas dopaminergic medication is primarily titrated in response to motor symptoms, it is increasingly understood that some cognitive impairments arise due to the effects of this therapy (Cools, 2006; MacDonald and Monchi, 2011). Clarifying the specific cognitive functions that are helped versus those that are hindered by dopaminergic medication can inform treatment in PD, allowing clinicians to consider cognitive as well as motor complaints in titrating therapy.

### Conflict of interest statement

The authors declare that the research was conducted in the absence of any commercial or financial relationships that could be construed as a potential conflict of interest.
